# Complications of Percutaneous Radiologic Gastrostomy Among Patients in a Tertiary Care Hospital in Riyadh, Saudi Arabia

**DOI:** 10.7759/cureus.38474

**Published:** 2023-05-03

**Authors:** Najla Alrasheed, Haneen S Khair, Renad M Aljohani, Noof M Alharbi, Nahlah N Alotaibi, Shahad F AlEdrees, Aamir Omair

**Affiliations:** 1 Department of Medicine, King Abdulaziz Medical City, Riyadh, SAU; 2 College of Medicine, King Saud Bin Abdulaziz University for Health Sciences, Riyadh, SAU; 3 Department of Internal Medicine, College of Medicine, King Saud Bin Abdulaziz University for Health Sciences, Riyadh, SAU; 4 Department of Research, King Abdulaziz Medical City, Riyadh, SAU; 5 Department of Medical Education/Research, King Saud Bin Abdulaziz University for Health Sciences, King Abdullah International Medical Research Center, Ministry of National Guard Health Affairs, Riyadh, SAU

**Keywords:** minor complications, major complications, prevalence, gastrostomy tube, percutaneous radiologic gastrostomy

## Abstract

Background: Percutaneous radiologic gastrostomy (PRG) is the method of choice for patients incapable of ingesting nutrition orally. The complications related to PRG are classified into major and minor complications. This article presents the prevalence of major and minor complications of PRG among adult patients admitted to King Abdulaziz Medical City (KAMC) in Riyadh, Saudi Arabia between 2017 and 2018.

Methods: This was a retrospective cross-sectional study, which included adult patients who underwent a new PRG intubation between 2017 and 2018 in KAMC in Riyadh, Saudi Arabia. The variables reviewed were the demographics, comorbidities, indications of tube insertion, major and minor complications, and mortality rates.

Results: A total of 105 patients who underwent PRG were covered in this study with a mean age of 69.2 + 20.4 years. The most common indications were neurogenic pharyngeal dysphagia (31%) and dementia (29%). Most of the complications reported were minor (40%) and major complications were found in 2%. The percentage of patients with both minor and major complications was 37%. The patients who had no complications made up 21%. Major skin complication was reported in 19 patients (18%), while leakage was the most occurring minor complication found in 49 patients (47%). The 30-day mortality was observed in five patients (5%) and one-year mortality was observed in 21 patients (20%), and none of them were related to the PRG tube.

Conclusion: This study found that the PRG procedure had low rates of complications in KAMC. The majority were minor complications, and the mortality rate was low with none being related to the tube itself. So PRG may be considered to be a relatively safe procedure.

## Introduction

Gastrostomy is a procedure that provides long-term enteral feeding for more than a month [1]. The main purpose of gastrostomy is to deliver sufficient nutritional support, hydration, and medications to individuals with conditions that lead to diminished oral intake [2]. Initially, gastrostomy was performed using invasive techniques. These are extremely invasive, and the risk of associated complications is high [3]. In 1980, gastrostomy tube insertion evolved to a less invasive method, which is percutaneous endoscopic gastrostomy (PEG) [4]. A year later, in 1981, the radiologic insertion of gastrostomy tubes emerged [5]. Percutaneous radiologic gastrostomy (PRG) is the least invasive of all three approaches. Also, it is associated with the least risk of post-procedure complications and has more favorable outcomes [3].

PRG is performed by interventional radiologists. Fluoroscopy guidance is used to determine the site of tube insertion in view of the anatomical relationships [6]. Generally, an antecedent to the PRG procedure is to terminate the use of certain drugs that increase the proneness of bleeding, such as anticoagulants, aspirin, or warfarin. Additionally, the PRG tube placement is preceded by ultrasound to assess the position of the stomach and any insinuating organs, mainly the margin of the left lobe of the liver [7].

Radiologically placed percutaneous gastrostomy tube is a somewhat efficient and low-risk procedure; however, it has some post-procedural complications [8]. According to the Society of Interventional Radiology, PRG complications are designated into major and minor complications. Major complications, such as aspiration, hemorrhage, and infection, require immediate intervention and an extended period of hospitalization [9]. One major complication is aspiration pneumonia, which occurs after aberrant access of fluids into the lower segment of the pulmonary system. It is considered a progressive infection, as it can lead to multiple complications, including acute respiratory distress syndrome, lung abscess, and respiratory failure, if not treated early [10]. The second major complication is hemorrhage, which is defined as an impairment of the blood vessels causing excessive blood loss [11]. Moreover, peritonitis is another major complication of PRG, which is an inflammation affecting the peritoneal cavity and is usually caused by leakage from the gastroduodenal tract into the peritoneal cavity [12]. In addition, minor complications commonly take place shortly after the tube insertion. They include obstruction, tube displacement, and skin-related conditions [1,8,9]. The obstruction could occur either in the lumen of the gastrostomy tube or in segments of the tube that can relocate in some parts of the gastric tract. Additionally, tube displacement could happen unintentionally in a period of one to two weeks after insertion [8]. If not discovered early, it can possibly induce peritonitis because of leakage of gastric components into the peritoneal space [1]. Furthermore, skin-related conditions that are associated with PRG are hypergranulation and superficial skin infections around the puncture site [3,9].

PRG is primarily indicated to replenish nutrients depleted in patients with dysphagia and anorexia caused by an underlying malignant condition, such as head and neck cancer (HNC), esophageal cancer, or neurological swallowing dysfunction and motility disturbance caused by cerebrovascular accidents (CVAs), neurodegenerative disorders such as amyotrophic lateral sclerosis or advanced dementia, or, less commonly, neuromuscular disorders such as myasthenia gravis and Guillain-Barre syndrome [1,13,14]. Among these, HNCs (especially oropharyngeal cancer) and esophageal cancer constitute the most common indications for PRG insertion, especially in advanced stages (American Joint Committee on Cancer (AJCC) stage III or beyond for HNCs), and 62% of pre-chemoradiotherapy esophageal cancer patients are in stage IV according to one study [7]. CVA-related dysphagia resolves with time, leaving only 2% of patients with permanent dysphagia who require PRG insertion [15]. Likewise, neuro-disability, anorexia, and decreased consciousness in dementia patients necessitate PRG intubation [16]. PRG placement for the purpose of reversal or alleviation of malnutrition accounts for 92% of all PRG cases [13,14]. The remaining cases are attributed to gastric decompression by means of a fluoroscopic gastrostomy to relieve symptoms of obstruction in patients with unresectable abdominal tumors leading to small bowel obstruction. Contraindications to the PRG procedure are mainly massive ascites in the infra-colic, gastric displacement due to an intrinsic abdominal tumor, or altered gastric anatomy following partial gastrectomy. Upon gastric insufflation, failure of the interposed colon to be dislodged may impede the placement of PRG and pose a risk of bowel perforation and subsequent peritonitis, making it a contraindication. Other anatomical barriers include hepatosplenomegaly and hiatal hernia [17].

This study aimed to measure the prevalence of PRG complications and identify the major and minor complications of PRG. Secondarily, the study aimed to identify the causes and risk factors of these complications. The study population included adult patients who had a PRG procedure at King Abdulaziz Medical City (KAMC) in Riyadh, Saudi Arabia, between 2017 and 2018. This study was intended to be informative to the interventional radiologists and internal medicine staff at KAMC about the possible complications of PRG that they may encounter.

## Materials and methods

Study design and area and settings

This was a cross-sectional study that was conducted by reviewing electronic medical records from BestCare (ezCaretech, Seoul, Korea) hospital information system. The electronic medical records were reviewed for all patients who received a PRG tube insertion between January 2017 and December 2018 at KAMC, a governmental tertiary care center located in Riyadh, Saudi Arabia. In KAMC, the majority of PRG tubes are inserted by interventional radiologists, but in a minority of cases, they are placed by gastroenterologists.

The sample was collected by including all patients who met the predefined inclusion criteria and who were admitted to KAMC in the selected period of our study using non-probability consecutive sampling. A population of about 1,000 was documented according to the medical unit in KAMC in the intended period of the study. An estimated sample size of 278 resulted when considering a 5% margin of error and 95% confidence interval by the Raosoft sample size calculator (Raosoft, Inc., Seattle, WA).

Identification of study participants

Patients who received a new PRG tube were considered for inclusion. Also, these patients had to be followed for one year for any complications to ensure that previous complications did not affect the outcomes. Only adult patients aged 18 years and up, both males and females of all nationalities, were included. The study excluded patients who had percutaneous endoscopic gastrostomy or nasogastric tubes since this study was directed toward the complications of the radiologically inserted tubes.

Data collection process

Data were collected by the co-authors using a data sheet. The selection of the data was dependent on the inclusion and exclusion criteria. The data were taken from the BestCare system in the Internal Medicine Department at KAMC. The data collection sheet was approved by the Institutional Review Board at the King Abdullah International Medical Research Center (KAIMRC). The data sheet was composed of nine categories. The "presenting comorbidity" category incorporated diabetes mellitus, hypertension, chronic kidney disease, heart failure, liver cirrhosis, a cerebral vascular accident, cancer, dementia, and malnutrition. In addition, the complications category was subdivided into major and minor complications. The major complications included peritonitis, internal hemorrhage, bowel perforation, abscess, and major skin complications. The minor complications included stomal infection, leakage, tube occlusion, and dislodgement. The data sheet included a category named "mortality during the first 30 days post-insertion," which had two questions. The first question was regarding the association between mortality and complications. If the complications were related to the mortality, then that needed to be specified. The last category was "mortality during one-year post-insertion." If mortality was declared, the period during which the tube was present was mentioned.

Data analysis

First, the Shapiro-Wilk test was applied to the whole dataset to assess univariate normality. After that, the mean and standard deviation were calculated for continuous variables in the demographical data (age and BMI). The data were entered into an Excel sheet (Microsoft Corporation, Redmond, WA) and analyzed by the Statistical Package for the Social Sciences (SPSS, IBM Corp., Armonk, NY). The demographics of the patients were analyzed using descriptive statistics. For the numerical data, including height, weight, and age, the mean and standard deviation were used. The categorical data were described as percentages and frequencies. The categorical data included the gender, presenting comorbidities, classification and severity of the complication, the reason for insertion, the mortality during the first 30 days (yes or no), and the mortality one-year post-insertion (yes or no). The chi-square test was used to identify the association between the utilization of PRG tubes and complications. A p-value < 0.05 was considered to show a significant association. Anonymity and confidentiality were maintained during this research. Patients’ names and medical record numbers were not used, and the data were kept confidential.

## Results

A total of 105 patients were included in the study, with 58 males (55%) and 47 females (45%), and the mean age was 69.2 + 20.4 years. Their mean body mass index was 23.2 + 5.7 kg/m^2^. The most common indications for PRG were nutritional support for neurogenic pharyngeal dysphagia (31%, n = 33), followed by dementia (29%, n = 30) and terminal cancer (13%, n = 14). Other indications consisted of feeding purposes for low oral intake, cerebral vascular disease, and paralysis (33%, n = 35), as presented in Table 1.

**Table 1:** Characteristics of the patients (N = 105)

		Mean	SD
Age		69.2	20.4
Body mass index (kg/m^2^)		23.2	5.7
		Number	%
Gender	Male	58	55%
	Female	47	45%
Indications			
Nutritional support for neurogenic pharyngeal dysphagia		33	31%
Nutritional support for elderly dementia and inadequate oral intake		30	29%
Terminal cancer patient		14	13%
Other		35	33%

The major complications following a PRG included major skin complications in 19 patients (18%), abscesses in 17 patients (16%), internal hemorrhage in nine patients (9%), and peritonitis in five patients (5%). For the minor complications, leakage was the highest occurring complication and was reported in 49 patients (47%), stomal infection was seen in 43 patients (41%), tube dislodgement in 39 patients (37%), and tube occlusion was observed in 20 patients (19%), as presented in Table 2.

**Table 2:** Complications after gastrostomy (N = 105)

		Number	%
Minor complications	Leakage	49	47%
	Stomal infection	43	41%
	Tube dislodgment	39	37%
	Tube occlusion	20	19%
	Other	5	5%
Major complications	Major skin complications	19	18%
	Abscess	17	16%
	Internal hemorrhage	9	9%
	Peritonitis	5	5%
	Other	2	2%

Patients with major skin complications, the commonest major complication, have a mean age of 77.1 + 22.1 years. The p-values of other major complications are all greater than 0.05. The mean age of the patients with the most common minor complication, which is leakage, was 70.7 + 22.0 years. All the p-values for minor complications were greater than 0.05, as presented in Table 3. For major complications, the mean of comorbidities was greatest in patients with post-insertion internal hemorrhage, with a value of 4.9 + 1.8. The mortality during the first 30 days post-insertion was 5% (n = 5), and the mortality during the first-year post-insertion was 20% (n = 21). However, none of the deaths were related to the PRG tube.

**Table 3:** Mean age of the patients with different complications (N = 105)

		Number	Mean	SD	p-value
Minor complications	Leakage	49	70.7	22.0	0.48
	Stomal infection	43	72.9	18.4	0.12
	Tube dislodgment	39	69.7	22.0	0.83
	Tube occlusion	20	75.1	18.5	0.15
	Other	5	58.0	23.9	0.21
Major complications	Major skin complications	19	77.1	22.6	0.06
	Abscess	17	73.6	21.4	0.33
	Internal hemorrhage	9	79.1	16.2	0.13
	Peritonitis	5	68.8	27.1	0.97
	Other	2	78.5	7.8	0.52

## Discussion

PRG is considered a relatively safe procedure with a high success rate according to several studies [2,7]. Despite it being safe, it has complications that are designated as major and minor, and in this study, the prevalence of these complications was assessed. In this research, the main indication for PRG insertion was neurogenic pharyngeal dysphagia in 31% of cases. In contrast to other research, neurological disease was the main indication in 59.6% of patients [18]. Compared to another study, tube insertion was applied to 41% of dementia patients, although this research noted tube insertion in 29% of dementia patients [2]. In previous studies, PRG was used in 38.3% of cancer patients, while in this study, it was indicated in 13% of terminal cancer cases [18].

The major skin complications were found to be the most common major complications, and the leakage was found to be the most common minor complication. Major skin complications included hypergranulation tissue, wounds, skin rash, and redness. According to this research, in KAMC, the incidence of minor complications (73%) was higher than the major complications (39%), like other studies in this aspect [19,20]. Major skin complications were found to be the most common major complication. These included hypergranulation tissue, wounds, skin rashes, and hypersensitivity reactions. The second most common major complication reported was an abscess (16%), in comparison to a study where the abscess was significantly lower (2.1%) [18]. For minor complications, in previous studies, leakage was recorded as one of the least occurring minor complications [18,20]. However, in the current study, leakage was found to be the most common minor complication that developed following a PRG. Following leakage, stomal infection was the second most common minor complication, in contrast to multiple studies where it was considered the most frequently reported minor complication [2].

In this study, age was not associated with the incidence of any of the major or minor complications of PRG. Furthermore, there were no significant differences between the presence of the comorbidities and the complication of PRG insertion detected, except for the internal hemorrhage group, which scored the highest mean of total comorbidities, indicating that the chance of encountering patients with the post-insertion internal hemorrhage who suffer from comorbidities is high. Insignificant results for the data are potentially dependent on our study limitations, which include an inadequate sample size. None of the mortalities were related to the procedure. Therefore, based on the results of this study and other literature, procedure-related mortality is low, and PRG is considered safe in clinical practice [1,21,22].

One of the limitations of this study was that the number of patients was less than the required sample size, although all patients who had PRG tubes were included. Another limitation is the inadequacy of data in the BestCare system, as the reports and notes were not thorough.

## Conclusions

In conclusion, PRG tube insertion is a relatively safe procedure, as indicated by the overall low rates of life-threatening events. It has major and minor complications, with a higher prevalence of minor complications. The total number of comorbidities is not a risk for any of the major complications, except for the internal hemorrhage, and none of the minor complications. Age is also not a risk factor for major or minor complications. Mortality is not directly related to PRG tube insertion. We recommend that more research be conducted on the prevalence of PRG complications with a larger sample size. Also, further studies focusing on the risk factors and success rates of the procedure are needed.
